# Supplementary figures and images for: Therapeutic targeting of ARID1A and PI3K/AKT pathway alterations in cholangiocarcinoma
Source: PeerJ. 2022 Jan 13;10:e12750. doi: 10.7717/peerj.12750 (PMC8761367; doi:10.7717/peerj.12750)

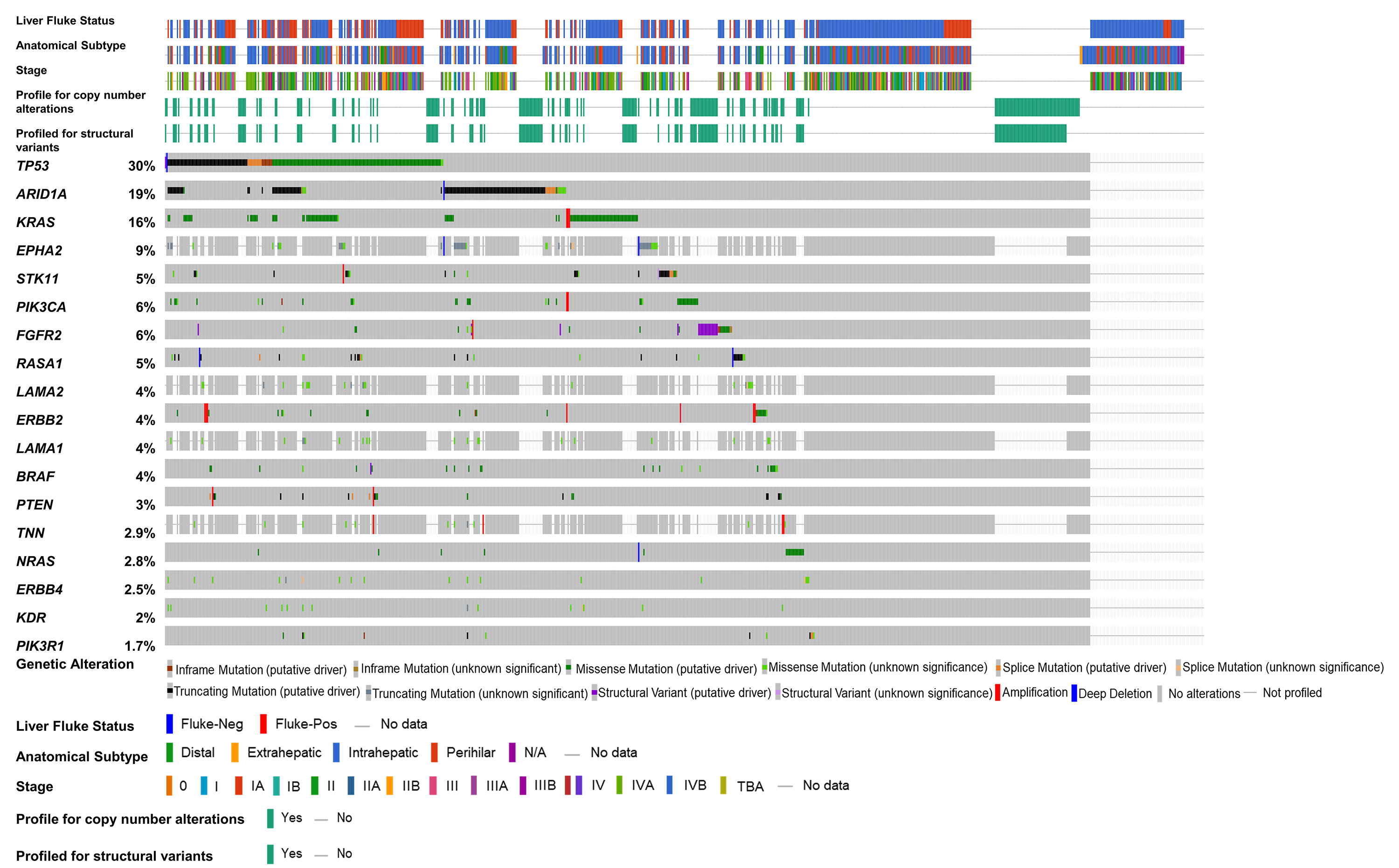

Supplement: Supplemental Information 1 — The Oncoplot was generated on cBioPortal (https://www.cbioportal.org). [file peerj-10-12750-s001.png]

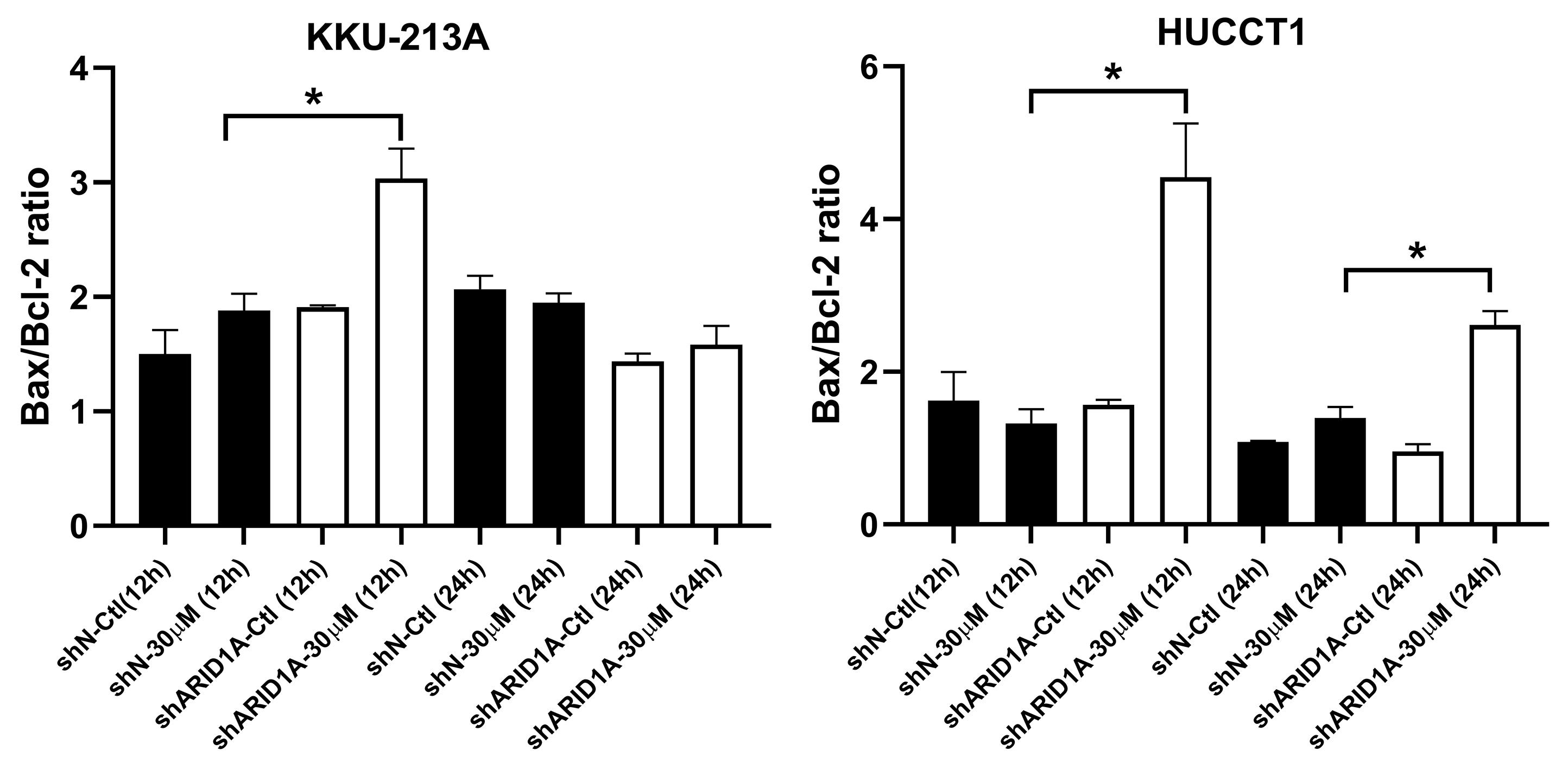

Supplement: Supplemental Information 2 — Relative Bax/Bcl-2 levels were increased in ARID1A-knockdown CCA cell lines compared to non-targeted shRNA control cells (shN). Densitometric quantification of the relative Bax/Bcl-2 expression in CCA cell lines were obtained using ImageJ (version 1.53a, NIH, USA). Cells were treated with MK-2206 for 12 h (12 h) or 24 h (24 h) and 0.3% DMSO was used as the control (Ctl). KKU-213A, ∗p = 0.032 and HUCCT1, ∗p = 0.025 and 0.018 respectively, unpaired t-test. [file peerj-10-12750-s002.png]
